# Editorial: POCUS for neonates - advancing care with point-of-care ultrasound

**DOI:** 10.3389/fped.2025.1721186

**Published:** 2025-10-28

**Authors:** Yogen Singh, Pradeep Suryawanshi, Belinda Chan

**Affiliations:** ^1^ Department of Pediatrics, Division of Neonatology, University of California, UC Davis Children's Hospital, Sacramento, CA, United States; ^2^ Department of Neonatology, Bharati Vidyapeeth (Deemed to be University) Medical College, Pune, India

**Keywords:** neonates, point-of-care ultrasound (POCUS), POCUS research, future directions, neonatal POCUS

**Editorial on the Research Topic**
POCUS for neonates: advancing care with point-of-care ultrasound

The clinical application of point-of-care ultrasound (POCUS) in neonatology has expanded dramatically over the recent decade, driven by the easy availability of portable machines and its proven value in making better physiology-based clinical decisions. POCUS has been demonstrated to be superior to chest x-rays and other bedside available tools in making accurate diagnoses, targeting specific interventions, and enhancing procedure safety. Its ability to perform serial evaluations helps in understanding the disease process and evaluating the response to intervention. Beyond diagnostics, POCUS enhances procedural success rates for central line placement, lumbar puncture, and thoracentesis, reducing complications compared to traditional “blind” techniques. Its radiation-free nature also makes it a safer alternative to CT scan or x-ray, particularly for newborns. These benefits make POCUS an attractive imaging tool in the intensive care unit. However, neonatal POCUS lags behind as compared to adult medicine. To promote a safe use of POCUS and enhance clinical governance, the European Society of Pediatric and Neonatal Intensive Care (ESPNIC) society issued the first international evidence-based guidelines on use of POCUS in neonatal and pediatric intensive care units (2020), followed by the American Academy of Pediatrics (AAP) clinical and technical reports (2022) (1, 2). There remains uncertainty about whether widespread adoption will improve the outcomes in the NICU.

This Research Topic, “*POCUS for Neonates: Advancing Care with Point-of-Care Ultrasound*,” brings together 10 peer-reviewed articles that enhance knowledge on how POCUS can be utilized for accurate and timely diagnosis, guide interventions, and improve decision-making in the NICU. Bhattacharjee et al. reported that repeated x-ray exposure was linked to developmental delay, underscoring the value of radiation-free imaging such as POCUS. Beyond being a reliable diagnostic and procedural tool (1, 2), Tomaszkiewicz et al. performed outcomes studies showing that POCUS impacts care by enhancing efficiency in umbilical venous catheter placement and reducing complications. LUS with qualitative scoring has been widely used for decades to predict surfactant need in preterm infants with respiratory distress syndrome, but standardized approaches remain lacking (3). Several studies here focused on LUS protocol standardization and application validation. Zaili and Na simplified and standardized LUS scoring by focusing on pulmonary consolidation with good predictive value for surfactant treatment. Chetan et al. validated that assessing 6 vs. 12 regions offered comparable predictive value for invasive ventilation. Chen et al. extended the use of LUS to detect consolidation not visible on chest x-rays, enabling personalized care through targeted broncholavage for airway clearance and ventilator adjustments for atelectasis. Quality assurance was emphasized by Singh et al., who found that using higher-frequency transducers improved the interpretability of abdominal ultrasound. They also emphasized the value of serial imaging and standardized reporting templates to enhance diagnostic reliability. Expanding into prognostication and risk-stratification roles, Dauengauer-Kirlienė et al. utilized cranial ultrasound to triage infants with lenticulostriate vasculopathy for genetic testing, identifying novel associations with WNK1 that may have potential long-term neurological implications. Song et al. found expanded applications for prenatal ultrasound in esophageal atresia subtyping, and Sohane et al. demonstrated the feasibility of liver POCUS in low-resource settings. Finally, Xing et al. highlighted that the advanced technology of contrast-enhanced ultrasound (CEUS)—widely used in adults for characterizing tumors and abscesses —can also improve the detection of small intestinal inflammatory myofibroblastic tumors in pediatric patients (4). As Singh et al. ways to improve the interpretability of AUS, CEUS may have the potential to enhance the diagnostic role in neonatal bowel injury in future research.

With the availability of ultra–high-frequency probes (UHFUS) (∼30–70 MHz), neonatal POCUS is less limited by the small size and superficial location of neonatal structures. UHFUS offers significantly higher spatial resolution for very superficial tissues (typical penetration of ∼1–3 cm) and has demonstrated feasibility in pediatric and neonatal imaging of lung, gastrointestinal, and musculoskeletal imaging (5, 6). As POCUS applications expand, research must also address effective methods for delivering high-quality and most efficient training for the clinicians.

The pace of innovation in technology used in the medical field, especially in imaging, is extremely fast. Application of Artificial Intelligence (AI) can help address operator dependence, variability in image quality, and challenges in quantitative analysis that characterize POCUS. The AI applications include better imaging optimization, help with the best possible imaging acquisition, rapid image analysis, and data processing (7, 8). Enhanced AI applications include real-time acquisition guidance that provides step-by-step feedback for probe placement and automatically stores optimal images (7, 8). This will help in POCUS training for new users, especially those in low-resource areas, which is another important POCUS research area. Other automated tools offer functions such as B-line counting for lung ultrasound scoring, inferior vena cava (IVC) compressibility assessment for intravascular volume status, velocity-time integral (VTI) calculations for cardiac output, and real-time ejection fraction (EF) measurements. Chan et al. developed an algorithm to automatically quantify the proportion of anechoic cerebrospinal fluid within the spinal canal to correlate with intraventricular hemorrhage severity (9). All these tools still require extensive validation due to both known and unknown limitations, particularly the inadequate neonatal data pool for machine learning.

Despite these advances, several limitations hinder the widespread clinical adoption of AI-enabled POCUS. Most models are trained on relatively small or homogeneous datasets, which limits generalizability across diverse patient populations, ultrasound machines, and clinical settings. The volume of neonatal imaging data available for machine learning remains insufficient, and many automated tools have not been adapted for neonatal physiology. Addressing these limitations through multicenter validation, transparent algorithm design, and thoughtful clinical integration will be critical for AI to achieve its potential in the field of ultrasonography.

**Figure 1:**
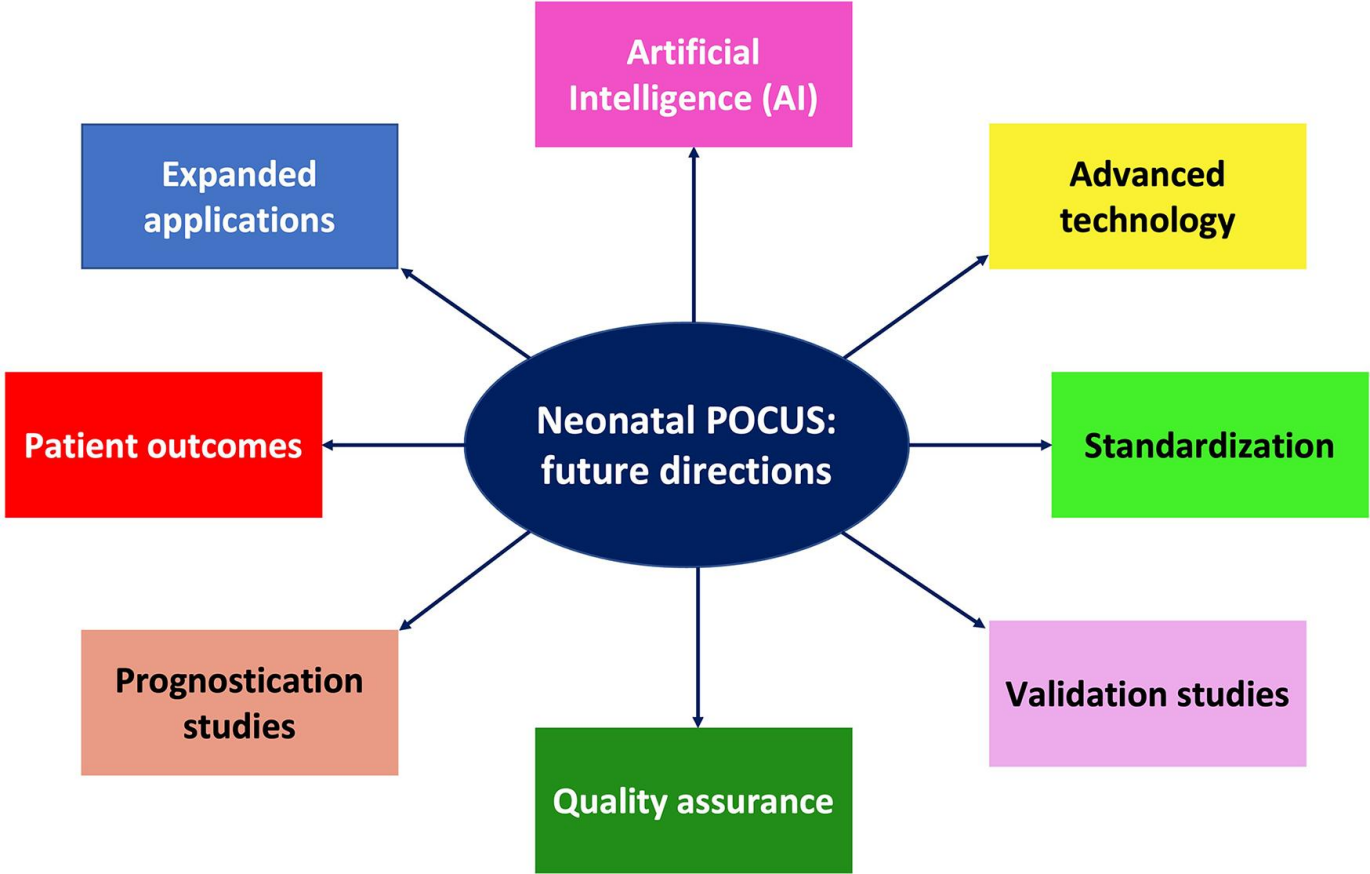
Schematic illustration of future directions in neonatal POCUS research and innovations for use in clinical practice.

In this editorial, we reflect on the rapidly evolving role of POCUS, some of the araes featured in this Research Topic, and outline future directions. POCUS is changing the way clinicians practice in the intensive care unit; however, continued investigation is essential to strengthen the body of evidence around its impact on improving patient outcomes. Future directions should include studies focused on patient outcomes, standardization of clinical applications, validation of guidelines and protocols, quality assurance, technological innovation, and how best to provide POCUS training (Figure 1).
